# Honey – a potential agent against *Porphyromonas gingivalis*: an in vitro study

**DOI:** 10.1186/1472-6831-14-24

**Published:** 2014-03-25

**Authors:** Sigrun Eick, Gesine Schäfer, Jakub Kwieciński, Julia Atrott, Thomas Henle, Wolfgang Pfister

**Affiliations:** 1 Department of Periodontology, Laboratory of Oral Microbiology, Dental School, University of Bern, Freiburgstrasse 7, CH-3010 Bern, Switzerland; 2 Medical University Laboratories, Institute of Medical Microbiology, University Hospital of Jena, Jena, Germany; 3 Department of Rheumatology and Inflammation Research, Sahlgrenska Academy, University of Gothenburg, Gothenburg, Sweden; 4 Institute of Food Chemistry, Technische Universität Dresden, Dresden, Germany

**Keywords:** Honey, *Porphyromonas gingivalis*, Biofilm, Methylglyoxal, Minimal inhibitory concentration

## Abstract

**Background:**

Honey has been discussed as a therapeutic option in wound healing since ancient time. It might be also an alternative to the commonly used antimicrobials in periodontitis treatment. The in-vitro study was aimed to determine the antimicrobial efficacy against *Porphyromonas gingivalis* as a major periodontopathogen*.*

**Methods:**

One Manuka and one domestic beekeeper honey have been selected for the study. As a screening, MICs of the honeys against 20 *P. gingivalis* strains were determined. Contents of methylglyoxal and hydrogen peroxide as the potential antimicrobial compounds were determined. These components (up to 100 mg/l), propolis (up to 200 mg/l) as well as the two honeys (up to 10% w/v) were tested against four *P. gingivalis* strains in planktonic growth and in a single-species biofilm.

**Results:**

2% of Manuka honey inhibited the growth of 50% of the planktonic *P. gingivalis*, the respective MIC_50_ of the German beekeeper honey was 5%. Manuka honey contained 1.87 mg/kg hydrogen peroxide and the domestic honey 3.74 mg/kg. The amount of methylglyoxal was found to be 2 mg/kg in the domestic honey and 982 mg/kg in the Manuka honey. MICs for hydrogen peroxide were 10 mg/l - 100 mg/l, for methylglyoxal 5 – 20 mg/l, and for propolis 20 mg/l – 200 mg/l. 10% of both types of honey inhibited the formation of *P. gingivalis* biofilms and reduced the numbers of viable bacteria within 42 h-old biofilms. Neither a total prevention of biofilm formation nor a complete eradication of a 42 h-old biofilm by any of the tested compounds and the honeys were found.

**Conclusions:**

Honey acts antibacterial against *P. gingivalis*. The observed pronounced effects of Manuka honey against planktonic bacteria but not within biofilm can be attributed to methylglyoxal as the characteristic antimicrobial component.

## Background

Periodontitis is a chronic inflammation that occurs in response to the presence of subgingival bacteria. A limited number of bacterial species have been associated with periodontitis, and strong evidence has been accumulated to implicate *Porphyromonas gingivalis*, an anaerobic gram-negative bacterium in the pathogenesis [1-3]. Moreover *P. gingivalis* was postulated to be a keystone pathogen in developing periodontal disease [4], virulence is most associated with its high proteolytic activity [5].

Based on the impact of pathogens, the antiinfective regimen is an important component in any treatment of periodontitis. In preventing recolonization of bacteria, chlorhexidine digluconate (CHX) is a widely used agent in periodontitis treatment [6,7]. Antibiotics are recommended for severe cases [8,9]. Development of resistance against antibiotics and side effects of the drugs implicate search for alternatives. Among others, plant-based therapy including the combination with antibiotics or the usage of honey might be one option [10,11].

Honey is an ancient wound treatment and was re-introduced into modern medical therapy because of its antimicrobial and wound-healing promoting efficacy [12]. Hydrogen peroxide was found to be an antibacterial constituent of honey [13]. For a couple of years special interest has been focused on the Manuka honey that derives from the Manuka tree (*Leptospermum scoparium*) growing in New Zealand. In the Manuka honey, having a high non peroxide activity, methylglyoxal was identified as the dominant antibacterial constituent [14]. Honey has been proven to be effective in treatment of recurrent herpes simplex lesions [15], burn wounds [16], postoperative infected wounds [17]. It was found to reduce numbers of mutans streptococci in saliva of xerostomic patients [18]. In patients with gingivitis and plaque, the Manuka honey was able to reduce bleeding and the amount of plaque [19].

Another important bee product having an antimicrobial activity is propolis, a resinous substance which bees use for sealing of their combs [20]. As an additive to toothpastes [21] or irrigation [22], propolis may promote periodontal health.

Our study was aimed to determine the effect of two types of honey and of their most known antimicrobial compounds hydrogen peroxide and methylglyoxal, as well as of propolis on *P. gingivalis* strains in planktonic growth and in a single-species biofilm.

## Methods

### *Porphyromonas gingivalis* strains

Twenty *P. gingivalis* strains from the strain collection of the Laboratory of Oral Microbiology (University of Bern, Department of Periodontology) were included in the screening experiments determining minimal inhibitory concentration (MIC) values of the honeys. Strains included were two laboratory strains (ATCC 33277, W83) and 18 banked as isolates from periodontitis samples (BGH40-2, D2-4-3, D5-2-2, J358-1, J361-1, J362-1, J374-1, J378-1, J424-1, J426-1, J430-1, J435-1, J439-1, M5-1-2, MaRL, PL55, PL110, PL126). In the follow-up experiments the type strain ATCC 33277 and three other strains (M5-1-2, MaRL, J361-1) were used. The selection was based on the different colony morphology. M5-1-2 strain forms smooth colonies, MaRL very rough ones and J361 colonies similar to those formed by the type strain. The identity was confirmed by 16S rDNA sequence analysis.

All strains were kept frozen prior to the experiments. They were transferred and cultured anaerobically (10% H_2_, 5% CO_2_, 85% N_2_) on Schaedler agar plates (Oxoid, Basingstoke, GB) containing 8% sheep blood 24 h before starting the experiments at 37°C.

### Honeys, their potentially antimicrobial compounds and propolis

A local (German) multifloral blossoms honey from a beekeeper and a New Zeeland Manuka honey (Manuka Health New Zeeland Ltd, Te Awamutu, New Zeeland) were selected for the experiments. Tests of culturing the two honeys in anaerobic conditions did not show any microbial growth; therefore no gamma irradiation was applied. All media in the experiments with honey contained 0.1% (v/v) Tween 20 to increase solubility of honey.

The content of hydrogen peroxide was measured by means of potassium permanganate [23] and those of methylglyoxal was determined by reverse-phase, high-performance liquid chromatography (RP-HLPC) according to Mavric et al. [14]. Methylglyoxal and hydrogen peroxide (both Sigma-Aldrich, Steinheim, Germany) were available as 40% and 30% solution in water. The propolis was obtained as a 20% ethanolic dilution from a local beekeeper. Thus, in all experiments testing propolis, the respective amount of ethanol was added.

### Determination of minimal inhibitory concentrations

The susceptibilities were determined by the micro-bouillon dilution technique being a standard technique in microbiology [24]. 17 parts (170 μl each) of Wilkins–Chalgren broth (Oxoid) were mixed with 1 part of bacterial suspension (10 μl) and 2 parts of the honey diluted in 0.9% sodium chloride (20 μl each). The final concentrations ranged between 1 and 10% (w/v) honey. Similarly, the MICs of methylglyoxal and hydrogen peroxide were determined. The concentrations to be tested were between 0.1 and 100 mg/l. The propolis was tested in the range of 20 – 20,000 mg/l. 0.9% sodium chloride solution served as growth control. After an incubation time of 42 h, the MICs were determined visually. The results were confirmed by subculturing of each 10 μl of broth on Schaedler agar plates. The MIC was defined as the lowest concentration repressing visible growth.

### Single-species biofilms

The bacterial strains were precultured in Schaedler broth (Oxoid) added by 8% lysed sheep blood overnight. To determine effects on a forming biofilm assay, slides were placed into wells of 24-well plates and have been covered with artificial saliva (250 μl each) for 1 h at 37°C to create a pellicle. 1 l of the saliva (ISO 10993) contained 0.7 g sodium chloride, 0.26 g disodium phosphate, 0.33 g potassium thiocyanate, 1.2 g potassium dihydrogen phosphate 1.5 g sodium hydrogen carbonate and 1.2 g potassium chloride; this solution was supplemented with 4 g porcine mucin type II and 50 g albumine. After removal of the artificial saliva, 1.8 ml of bacterial suspension was transferred to the wells, followed by 200 μl of honey in different dilutions. The final concentrations of honey in the mixtures were 1 and 10% (w/v). The negative control was 0.9% sodium chloride solution. After 6 h and 24 h of incubation in an anaerobic atmosphere at 37°C, the slides were removed, shortly dipped into 0.9% sodium chloride solution to remove non adherent bacteria and then placed into other tubes containing 0.9% sodium chloride solution. The tubes have been exposed to ultrasonication of 160 W (Sonorex Super RK102H, Bandelin, Berlin, Germany) for 1 min. After a subsequent vortexing for 1 min, the numbers of viable *P. gingivalis* were determined as colony forming units (CFU) after plating of 100 μl of the suspension on Schaedler agar plates and cultivation.

Further, the effects of the honeys on a 42 h-old biofilm were tested. In these experiments, the bacterial suspensions were placed into the wells after creating the artificial pellicle. Forty-two hours after starting incubation, the supernatants were carefully removed and replaced by nutrient broth and honey solutions. The final concentrations used in these experiments were also 1% and 10% (w/v) honey. After an additional incubation time of 6 h and 24 h, the numbers of viable bacteria were determined as described above.

In follow-up experiments, hydrogen peroxide and methylglyoxal were tested instead of honey. The final concentrations were 5, 20 and 100 mg/l hydrogen peroxide and methylglyoxal respectively. The propolis was tested in the final concentrations of 20 mg/l and 200 mg/l.

All experiments were made in three independent repetitions at least. The statistical analysis was made by Student’s t-test for independent samples by using SPSS Statistics v.17.0 (IBM, Chicago, IL). Test samples were each compared with controls. The level of significance was set to p < 0.05.

## Results

### Content of potentially antimicrobial compounds

The Manuka honey contained 1.87 mg/kg hydrogen peroxide and the domestic honey 3.74 mg/kg. The amount of methylglyoxal was found to be 2 mg/kg in the domestic honey and 982 mg/kg in the Manuka honey.

### Minimal inhibitory concentrations

In the screening assays including 20 *P. gingivalis* strains, the minimal inhibitory concentration against 50% of the included strains (MIC_50_) was 5% for the local domestic beekeeper honey and 2% for the Manuka honey. The domestic honey did not inhibit the growth of three strains up to 10% of the honey, whereas the growth of only one strain was not suppressed by 10% (w/v) Manuka honey.

In continuing experiments four strains were included. The growth of *P. gingivalis* strains was inhibited by 10 mg/l of methylglyoxal with the exception of the reference strain (ATCC 33277) where the MIC was 100 mg/l of the compound. The range of the MICs of hydrogen peroxide was between 5 and 20 mg/l. The propolis was growth-inhibitory in the range of 20 mg/l (M5-1-2 strain) to 200 mg/l (MaRL strain) (Table 1).

**Table 1:** **Minimal inhibitory concentrations (MIC) of honeys, their potential antimicrobial components and propolis against four *Porphyromonas gingivalis *** **strains**

***Porphyromonas gingivalis *** **strain**	**MIC of honeys (% w/v)**		**MIC of components (mg/l)**		**MIC of propolis (mg/l)**
	
	**Manuka**	**Local**	**Hydrogen peroxide methylglyoxal**		
ATCC 33277	2	5	10	100	40
M5-1-2	2	5	5	10	20
MaRL	2	10	20	10	200
J361-1	2	5	5	10	40

### Effects of honey on biofilms

**Figure 1:**
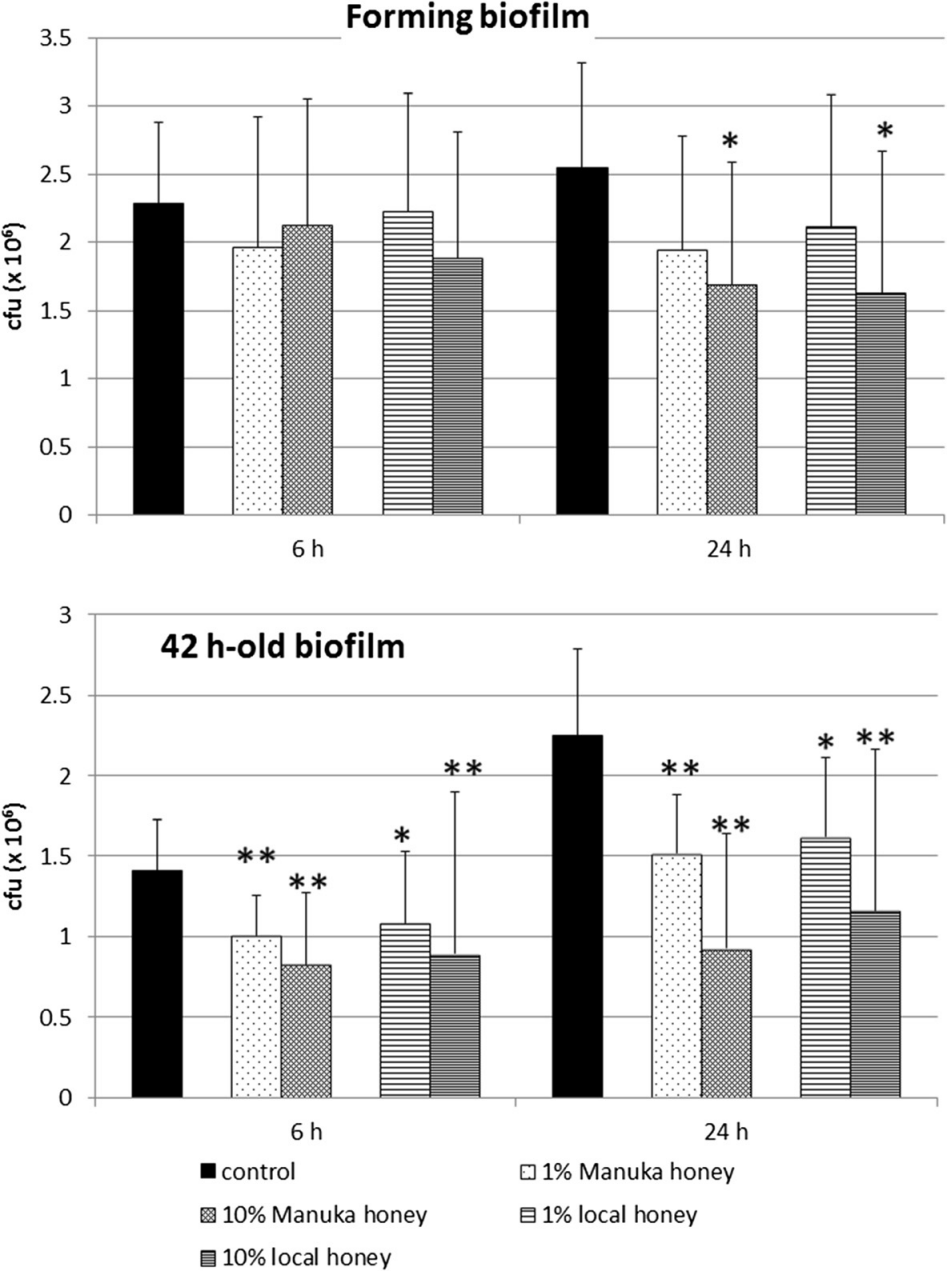
**Effect of honey on formation as well as on a 42 h-old single biofilms of four *Porphyromonas gingivalis *strains.** Manuka honey and a German local honey were added in two concentrations at the beginning of formation or on an already 42-h old biofilm. Colony forming units were determined each 6 h and 24 h after addition of the honeys (CFU counts within biofilm; *p < 0.05; **p < 0.01 compared to controls at the respective time).

Honey inhibited the formation of *P. gingivalis* single-species biofilm. Six hours after starting experiments, no differences were visible. At the 24 h time, both kinds of honey reduced concentration-dependently the numbers of viable bacteria. When the higher concentration of 10% was used, the numbers of bacteria in biofilm (mean of all strains) were significantly lower (each p < 0.05) than in the controls without addition of honey (Figure 1).

Addition of the honeys to an 42 h-old biofilm resulted also in a reduction of the mean counts of *P. gingivalis* strains within the biofilm 6 h and 24 h after exposure (6 h 1% locally produced honey p < 0.05, all others p < 0.01). The differences between the two types of honey were never significant within the same concentration neither in the experiments testing the effect on biofilm formation nor if a 42 h-old biofilm was studied (Figure 1).

**Figure 2:**
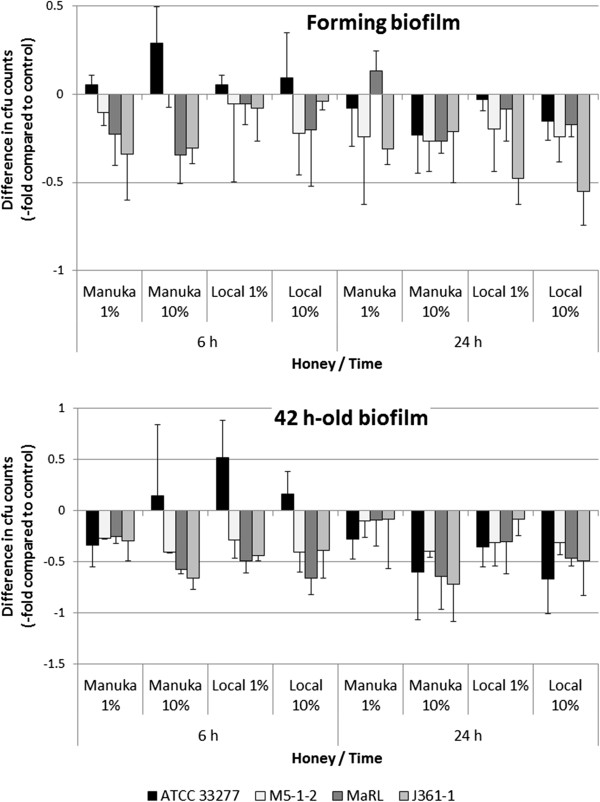
**Relations of single *Porphyromonas gingivalis *** **biofilms after addition of honeys to untreated controls each (colony forming units).**

When analyzing the strain dependent effects, the clinical isolates were more susceptible in comparison with the type strain (control). The differences were significant (p < 0.05) for 1% (w/v) of Manuka honey 6 h after starting biofilm formation. In the experiments testing the effects of honeys on 42 h-old biofilms, 10% of the Manuka honey (p < 0.05) as well as 1% and 10% of the locally produced honey (each p < 0.01) showed a stronger antibacterial efficacy on clinical isolates than on the reference strain 6 h after addition to the existing biofilms. 24 h after addition of the honeys to both types of the experiments, strain dependent differences were not visible any longer (Figure 2).

### Effects of methylglyoxal and hydrogen peroxide on biofilms

**Figure 3:**
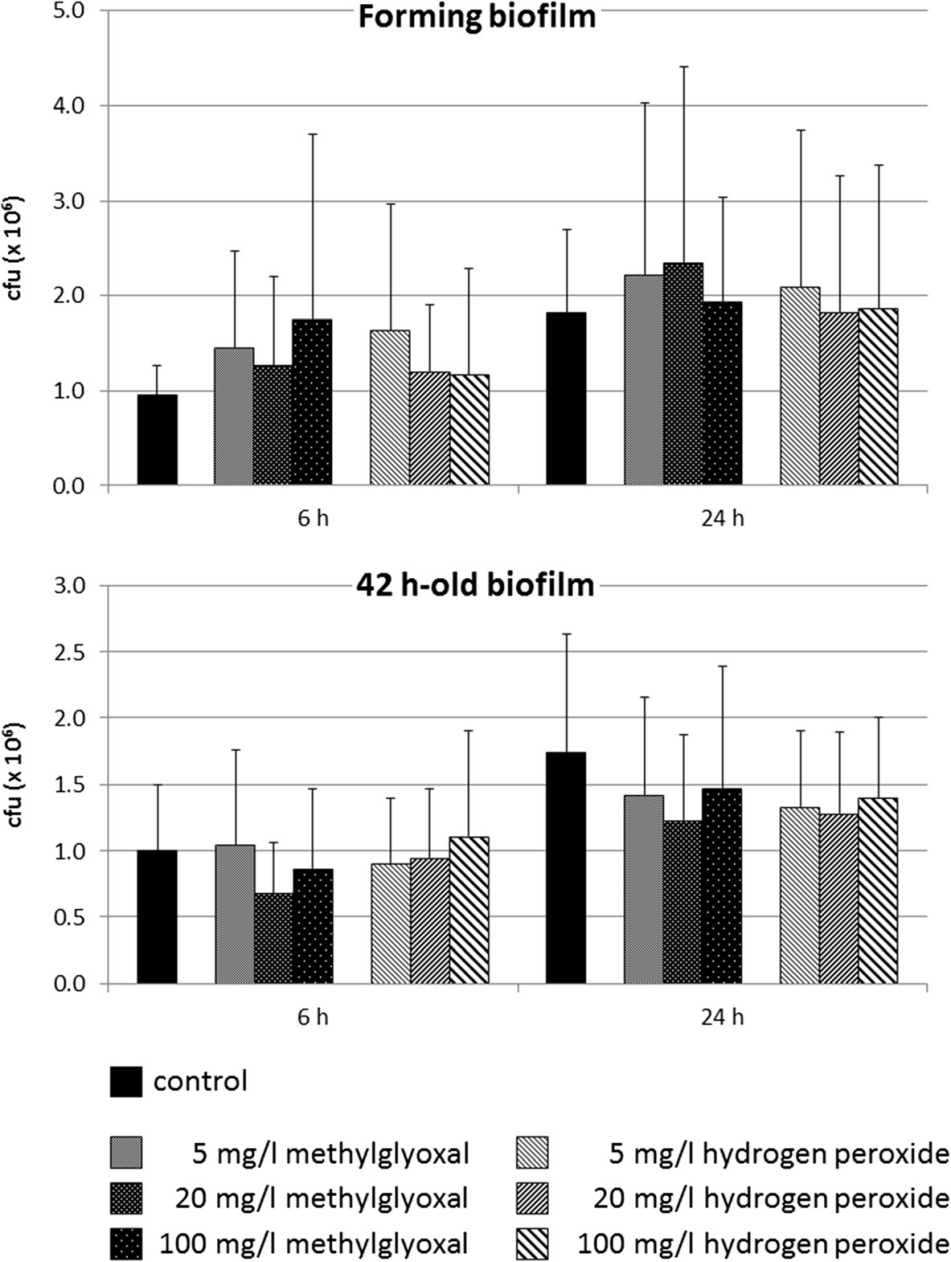
**Effect of potential antimicrobial compounds of honey on formation as well as on a 42 h-old existing single-species biofilms of four *Porphyromonas gingivalis *strains.** Methylglyoxal being the potential antimicrobial compound of Manuka honey and hydrogen peroxide as the potential antimicrobial compound of local German honey were added in three concentrations at the beginning of formation or on an already 42-h old biofilm. Colony forming units were determined each 6 h and 24 h after addition of the compounds (CFU counts within biofilm; *p < 0.05; **p < 0.01 each compared to controls at the respective time).

The addition of the antibacterial compounds methylglyoxal and hydrogen peroxide did not change the CFU counts of *P. gingivalis* within biofilm at any time point, neither in the biofilm-forming assays nor in the experiments using 42 h-old biofilms (Figure 3). Further, strain dependent differences were not detected (data not shown).

### Effect of propolis on biofilms

**Figure 4:**
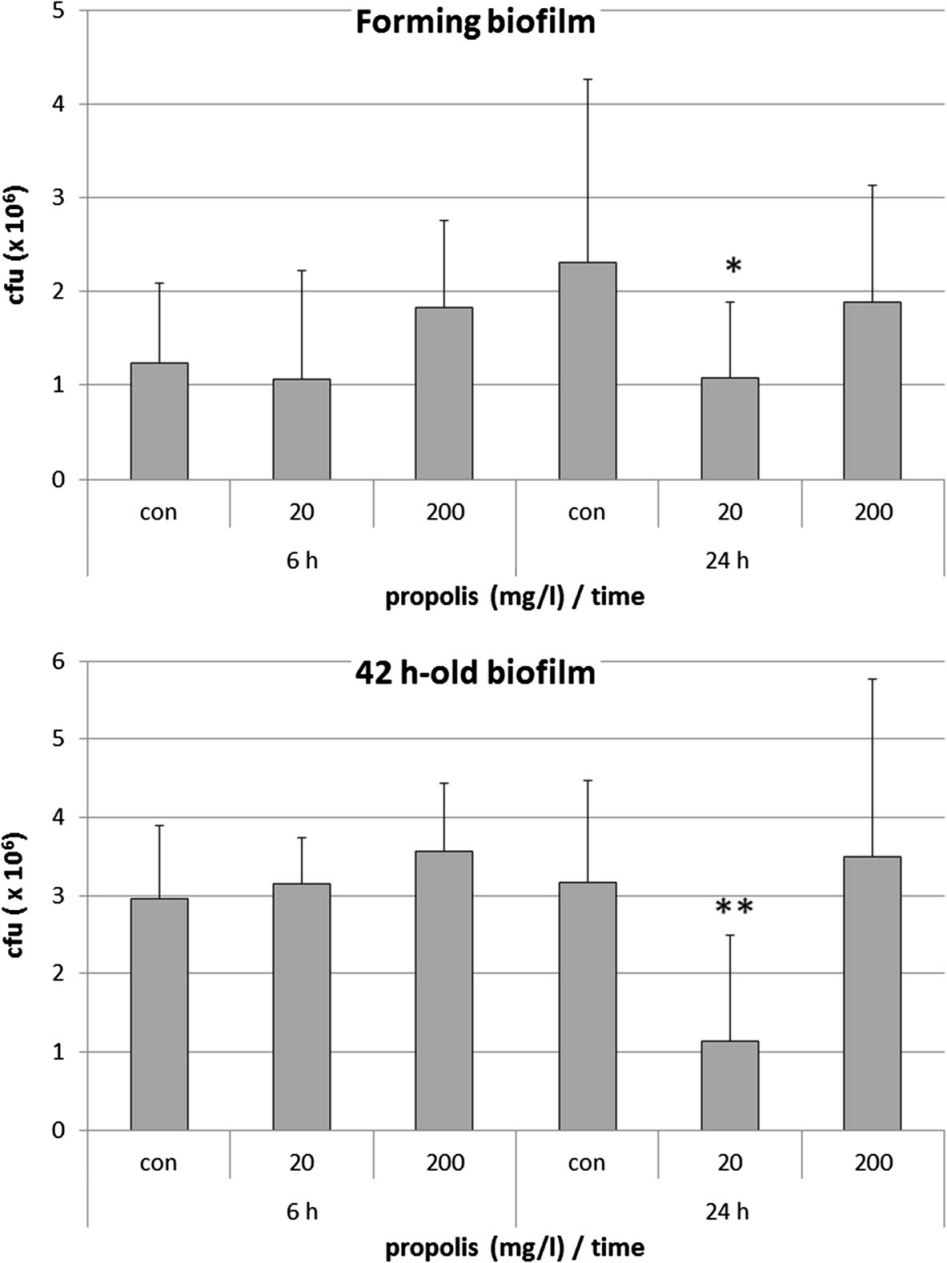
**Effect of propolis on formation as well as on a 42 h-old single-species biofilms of four *Porphyromonas gingivalis *strains.** Propolis was added in two concentrations at the beginning of formation or on an already 42-h old biofilm. Colony forming units were determined each 6 h and 24 h after addition of propolis (CFU counts within biofilm; *p < 0.05; **p < 0.01 each compared to controls at the respective time).

A propolis concentration of 20 mg/l reduced the CFU counts in the forming biofilm after 24 h (p < 0.05). No effects were found by testing the higher concentration of 200 mg/l. Addition of the propolis to a 42 h-old biofilm did not change the numbers of bacteria after 6 h. After 24 h the lower concentration of 20 mg/l propolis was effective in reducing the CFU counts; again, 200 mg/l did not show any effect (Figure 4). The most resistant strain was the MaRL strain; in the forming biofilm as well in the 42 h-old biofilm, higher CFU counts were found in comparison to the other strains after 24 h addition of 200 mg/l propolis (data not shown).

## Discussion

Honey acts antibacterial against *P. gingivalis* strains, the effect is being more pronounced for the Manuka honey compared to a locally produced beekeeper honey. The obtained MIC values are in the range or below those reported for other species. *E.g*., 10 – 50% honey were growth inhibitory against several enterobacteriacae and staphylococci, different kinds of honey showed only low differences in antimicrobial efficacy [25-27]. Contradictory are the results concerning oral microbes. In one study MICs of 12.5 - 25% were determined against oral streptococci [25], whereas others found 0.1% growth-inhibitory against oral streptococci and anaerobes [28].

Honey contains different antimicrobial compounds. In this study, hydrogen peroxide and methylglyoxal were separately tested. The content of methylglyoxal within Manuka honey was nearly 1 g/kg, slightly higher than determined by others [29,30]. Following from the MICs of the honeys and methylglyoxal, this compound might be responsible for the antimicrobial activity of the Manuka honey against the planktonic *P. gingivalis* strains. The hydrogen peroxide content was higher in the domestic honey than in the Manuka honey. Others did not find any hydrogen peroxide within Manuka honey [30]. But another study where antimicrobial efficacy was decreased when hydrogen peroxide was removed from Manuka honey implicates also a content of that substance within honey. Our results obtained by a widely used method might be false positively influenced by organic compounds of the honey [23]. Nevertheless, the measured concentrations of hydrogen peroxide within both types of honey were much too low for an antimicrobial action against the *P. gingivalis* strains included in our study. But interestingly, the strain exhibiting the most resistance against hydrogen peroxide was also least sensitive to the locally produced honey. Another not studied potential antimicrobial compound in the honey is the sugar but it exerts no or limited antibacterial affect [25,26]. Moreover, naturally occurring antimicrobial peptides might contribute to the antimicrobial efficacy of the honeys, recently a bee defensin has been discovered in a Medical (Revamil® source) honey [30]. Further, an effect of a decreasing pH cannot be completely excluded, as 10% of honey reduced the pH by about pH 0.3 as measured in broth. Propolis acts antibacterial against different oral anaerobes among them *P. gingivalis*[31]; MICs of propolis against *P. gingivalis* ATCC 33277 were reported in two studies as being between 64 and 512 mg/l (different kinds of propolis originating from Brazil and Turkey have been tested) [32,33]. In our study, only one kind of propolis originating from a lowland region in Germany characterized by a temperate climate was included. MIC against *P. gingivalis* ATCC 33277 was found to be slightly lower than reported before. MICs against the clinical isolates were not unique; the range was 20 – 200 mg/l.

Biofilms are known to be more resistant against antimicrobials than planktonic growing bacteria; antibiotics are 100-fold less sensitive against *P. gingivalis* in biofilm [34]. Therefore, honeys including its potentially antimicrobial compounds were also tested on a *P. gingivalis* biofilm. The used model was a simple one using artificial saliva to simulate oral conditions. But it has to be noted that dental plaque represents a much more complex highly organized biofilm [35,36] consisting up to 7000 species-level phylotypa [37], where communication occurs within different species [38]. Here, we wanted to show a potential effect on a forming biofilm representing the in vivo situation after mechanical disruption of a biofilm and on a 42 h-old biofilm. The efficacy of honey and its compounds on *P. gingivalis* biofilms was limited. Neither a complete eradication of a 42 h-old biofilm nor a total prevention of a biofilm formation was shown. Only 10% of both kinds of honey as well as 20 mg/l propolis were able to reduce the CFU counts within biofilm 24 h after beginning of biofilm formation. Surprisingly, the effects of honey were more remarkable on a 42 h-old biofilm. In our study the action of especially the Manuka honey against *P. gingivalis* biofilms seems to be not only due to a bactericidal effect of methylglyoxal as is was itself non effective under those conditions. A potential interaction with *P. gingivalis* capsule may play a role in disrupting biofilms suggested by the finding that in contrast to most clinical isolates the most resistant ATCC 33277 strain is characterized by missing capsule formation [39,40]. Recently, it was reported that about 25 – 50% of honey were bactericidal to *S. aureus* including methicillin-resistant strains and *Pseudomonas aeruginosa* overnight single-species biofilm, contrary to most of the antibiotics that did not show any effect [41]. Another study found 6 – 12% of Manuka honey and 12 – 25% of a Norwegian forest honey preventive in biofilm formation of staphylococci and gram-negative aerobes [42]. Only one study examined the effect of methylglyoxal on biofilm formation, 1.05 mg/ml methylglyoxal were bactericidal against 30 h-old *S. aureus* biofilms [29]. Interestingly, this value was also higher than those measured in its respective honey [29] implicating that methylglyoxal is not the only compound acting bactericidal. In these studies the tested concentrations of honey and methylglyoxal were higher than in ours assays. We had to ensure suitable growth conditions of *P. gingivalis* which required a sufficient amount of nutrient media. Further in oral cavity, a dilution effect by saliva and gingival crevicular flow has to be considered. Nevertheless, subsequent studies should test also higher concentrations of methylglyoxal.

In experiments using propolis, an inhibitory effect only in the concentration of 20 mg/l was found, the higher tested concentration of propolis did not have any effect on the counts of viable bacteria within biofilms. Probably the limited effect of propolis is not only due to the direct antimicrobial actions. Propolis negatively interacts with *S. aureus* adhesion and biofilm formation by inhibiting virulence factors in low concentration [43].

In addition to its antibacterial activity, immunmodulatory effects of honey have been described. Honey stimulates the release of inflammatory cytokines from monocytes [44], mRNA expression of TGF-β as an wound-healing promoting cytokine is upregulated [45]. Propolis does not have a high cytotoxicity against periodontal ligament cells [46]. It suppresses syntheses of inflammatory cytokines, whereas TGF-β1 is increased [47].

In an in vitro study it was shown that chlorhexidine acts more antibacterial than honey [48]. However, the addition of honey or its components as natural products to mouth rinses, tooth pastes may be discussed as a beneficial option in prevention and therapy of periodontitis. A subgingival irrigation with propolis extraxt improved the clinical parameters and reduced the counts of *P. gingivalis*[22]. In a pilot study including gingivitis patients, the usage of a chewing gum containing Manuka honey reduced the inflammatory variables and the plaque-score in comparison to a chewing gum without honey [19].

## Conclusions

In conclusion, honey especially Manuka honey acts growth-inhibitory on *P. gingivalis* as a major periodontopathogen*.* This effect on planktonic bacteria but not within biofilm is based on the ingredient methylglyoxal. Honey may destroy biofilms containing *P. gingivalis*. Therefore an addition of honey or its compounds to oral health-care products may have potential in prevention and treatment of periodontitis. More studies are needed to verify specifically the effect of honey and its compounds on oral species associated with periodontitis.

## Competing interests

The authors declare that they have no competing interests.

## Authors’ contributions

SE participated in planning and designing the study as well as in the data analysis. GS performed the microbiological laboratory work. JK participated in study design and data analysis. JA and TH performed analysis of the honeys. WP participated in planning and designing the study. All authors participated in drafting the manuscript; they have read and approved the final manuscript.

## Pre-publication history

The pre-publication history for this paper can be accessed here:

http://www.biomedcentral.com/1472-6831/14/24/prepub
